# Research trends on nanomaterials in gastric cancer: a bibliometric analysis from 2004 to 2023

**DOI:** 10.1186/s12951-023-02033-8

**Published:** 2023-08-02

**Authors:** Li-Xiang Ling, Yaobin Ouyang, Yi Hu

**Affiliations:** 1 Department of Gastroenterology, Digestive Disease Hospital, The First Affiliated Hospital of Nanchang University, 17 Yong Waizheng Street, Donghu District, Nanchang, 330006 Jiangxi Province China; 2 Department of Oncology, Mayo Clinic, Rochester, MN USA; 3 Department of Surgery at the Sir YK Pao Centre for Cancer, The Chinese University of Hong Kong, Shatin NT, Hong Kong, China

**Keywords:** Bibliometric analysis, Gastric cancer, Nanomaterial, Research trend

## Abstract

**Background:**

Gastric cancer is one of the leading causes of cancer-related deaths worldwide. In recent years, an increasing number of studies aimed at designing and developing nanomaterials for use in diagnosing and treating gastric cancer have been conducted. In this study, we aimed to comprehensively assess the current status and trends of the research on the application of nanomaterials in gastric cancer through a bibliometric analysis.

**Methods:**

Studies focusing on nanomaterials and gastric cancer were retrieved from the Web of Science Core Collection database and relevant articles were selected for inclusion in the study according to the inclusion criteria. Bibliometric and visual analysis of the included publications was performed using VOSviewer and CiteSpace.

**Results:**

A total of 793 studies were included. An increase in annual publications was observed from 2004 to 2023. China, Iran and the USA were the dominant countries in this field, accounting for 66.1%, 11.5% and 7.2% of publications, respectively. Shanghai Jiao Tong University and Cui DX were the most influential institution and author, respectively. The International Journal of Nanomedicine was the most prolific journal; Biomaterials was the most cited and most cocited journal. Nanomaterial-related drug delivery and anticancer mechanisms were found to be the most widely researched aspects, and green synthesis and anticancer mechanisms are recent research hotspots.

**Conclusion:**

In this study, we summarized the characteristics of publications and identified the most influential countries, institutions, authors, journals, hot topics and trends regarding the application of nanomaterials in gastric cancer.

**Graphical Abstract:**

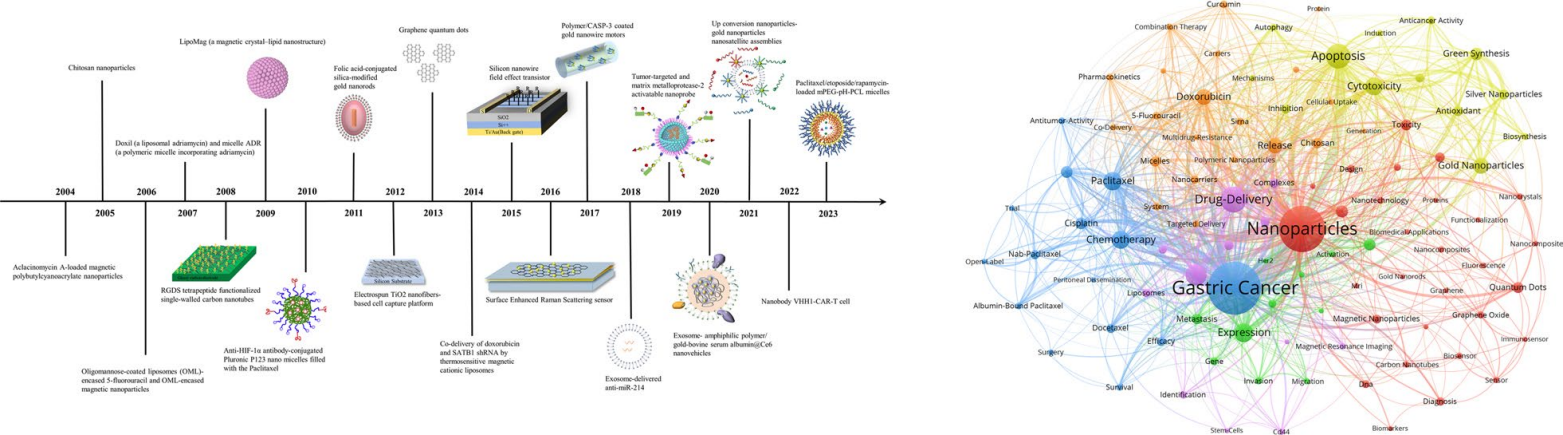

## Introduction

Gastric cancer (GC) remains the fifth most commonly diagnosed cancer and the fourth leading cause of cancer deaths, with over 1,000,000 new cases and an estimated 769,000 deaths occurring in 2020 [1]. Currently, the main challenge in diagnosing GC is the limited sensitivity of the methods available to detect small lesions in the early stages or after radiotherapy and chemotherapy. Moreover, the markers for diagnosing GC achieved unsatisfactory efficacy [2]. Despite the combination of treatment modalities used in the treatment of GC, such as biological agents in combination with chemotherapy to inhibit tumor progression and recurrence, the poor target and affinity of the drugs resulted in low therapeutic efficacy and serious side effects for patients. As such, novel and efficient methods are needed to diagnose and treat GC.

Nanomaterials, which have been extensively researched in the accurate diagnosis and efficient treatment of cancer due to their unique properties, are typically defined as materials with diameters ranging from 1 to 100 nm [3]. As detection and imaging agents, novel nanomaterials realize early diagnosis and precise positioning of tumors or diseased tissues, thereby improving the defects of traditional clinical detection and imaging agents [4, 5]. Meanwhile, nanomaterials can be used as drug carriers to achieve targeted and precise delivery of drugs, reducing side effects and drug resistance [6]. In addition, due to their unique physical properties, nanomaterials can realize photothermal/photodynamic, acoustic dynamic, magnetothermal therapy and combined therapy of tumors. Autophagy appears to be beneficial due to its tumor-suppressive effects, and this mechanism may be activated when engineered nanomaterials are introduced into cells; therefore, therapeutic interventions using nanoparticles (NPs) to modulate autophagy in malignant cells are likely to sensitize cancer cells to certain therapeutic modalities (e.g., radiation therapy) [7]. Sargazi et al. [8] developed F127/cisplatin microemulsions and found that low doses of F127/cisplatin microemulsions had less toxic effects on rat tissues but no increased cytotoxicity against malignant cells than that of free cisplatin. Titanium dioxide nanoparticles (TiO_2_ NPs) have tremendous photocatalytic activity and are promising materials for oncological photodynamic therapy and photothermal therapy. However, the wide bandgap of TiO_2_ limits its absorption to the ultraviolet spectrum only, rather than the near-infrared light region [9]. Consequently, the TiO_2_ NPs should be modified to allow them to respond to near-infrared light to achieve photocatalytic treatment, and the modified TiO_2_ has better biological safety and degradation [9].

Aptamer-functionalized carbon-based nanomaterials have been used as nanovesicles for targeted delivery of anticancer agents (e.g., doxorubicin and 5-fluorouracil) to the tumor site; however, carbon-based nanomaterials aggregate to form compounds that accumulate in vivo and lead to toxic effects; therefore, desirable interactions between functionalized carbon-based nanomaterials and plasma proteins need to be guaranteed [10]. Nanoghosts contain molecules from the surfaces of normal cells, maintain the targeting mechanism of progenitor cells, escape the immune system, remain in the circulatory system for a longer period and have been widely applied in tumor targeting [11]. Several newly designed nanostructures, such as immunostimulatory nanoadjuvants, liposome-based vaccines, polymeric vaccines, virus-like particles, lipid/calcium/phosphate NPs, and chitosan-derived nanostructures, have been used to deliver molecular, cellular, or subcellular vaccines to breast cancer cells to increase the efficacy and persistence of antitumor immunity while minimizing adverse side effects [12]. The main reason that nanostructures have not enhanced clinical practice in breast cancer as expected may be the lack of adequate preclinical models to effectively simulate actual breast cancer and its intricate interactions with the surrounding microenvironment, both spatially and physiologically [13]. The use of nanomaterials in the diagnosis and treatment of cancers is promising, but there are many challenges to be overcome before clinical application can occur.

Bibliometric analysis is a statistical method based on public literature databases that can provide a quantitative and qualitative evaluation of publications to aid in the analysis of research hotspots and trends in a specific field [14]. Pei et al. [15] assessed the current perspectives and trends in the research of nanomedicine in cancer from 2000 to 2021 using bibliometric analysis and summarized the development prospects and challenges faced by the application of nanomedicine to cancer treatment. Mahdieh et al. [16] analyzed research trends in magnetically functionalized nanoparticles for the treatment of colorectal cancer. To our knowledge, no bibliometric analysis has been published focusing on the application of nanomaterials in GC treatment. In this study, we aimed to use a quantitative approach to analyze the application of nanomaterials in GC treatment, identify the main contributors and the current status of the research in the field, and propose future research trends.

## Methods

### Search strategy

The Web of Science Core Collection (WoSCC) database was used to identify all relevant publications. It contains a large number of scientific publications and provides a source of general statistics for bibliometrics software; thus, it is the most frequently used database in bibliometrics research [14, 15]. All studies until July 16, 2023 were retrieved and downloaded from the WoSCC database. The search strategy to obtain articles on nanomaterials and GC involved using ((Gastric OR Stomach) AND (cancer OR tumor OR carcinoma OR neoplasm OR tumorous OR neoplastic)) AND (nano*). The search phrase "nano*" was used to find all terms beginning with "nano," including nanoparticles, nanomaterials, nanocarriers, nanocomposites, nanotechnology, etc.

### Study selection

All relevant publications were assessed in two stages by two authors (Li-Xiang Ling and Yao-bin Ouyang) independently, and every disagreement was thoroughly discussed with the third author (Yi Hu). At the first stage of screening, the language of the publications was restricted to English. In addition, nonarticle studies (reviews, conference proceedings, letters, etc.) were excluded from our study. At the second stage, the titles and abstracts of the remaining studies were carefully evaluated according to the following criteria: P (patient): the study involved GC patients, GC animal models and GC cell models; I (intervention): application of nanomaterials; and S (study design): clinical and basic research.

### Data extraction

The included publications were divided into different file formats for analysis. The following data were extracted from the included publications: title, author, institution, country, journal (including the journal impact factor (IF) of 2022), publication year, citation number and H-index.

### Data analysis

The included publications and cited references were exported as plain text for bibliometric analysis and visualization. VOSviewer (version 1.6.19), CiteSpace (version 6.2. R4) and GraphPad Prism (version 9.5.1) were used to generate visual graphs. GraphPad Prism was used to generate line graphs of the number of publications, citations and H-index for each year. VOSviewer was used to create visual graphs and analyze the most prolific/cooperative countries, institutions and authors, as well as the most cocited journals and most cooccurring keywords. CiteSpace was used to construct the timeline graph and bursts of keyword terms. Each dot on the visual graphs represents a country, institution, author or journal, and these dots were clustered into different groups according to their cooperation. The size of the dot was dependent on the number of publications. Link strength (LS) was the thickness of the line connecting the nodes and represented the strength of cooperation between them, and total link strength (TLS) reflected the overall level of cooperation. [15] In the keyword analysis, several meaningless keywords were excluded, and keywords with the same meaning were merged to obtain a better perspective. The modularity value (Q-value) > 0.3 and mean silhouette value (S-value) > 0.7 of the graphs generated by CiteSpace indicate significant and reasonable clustering. [17]

## Results

### Study selection and characteristics

**Fig. 1:**
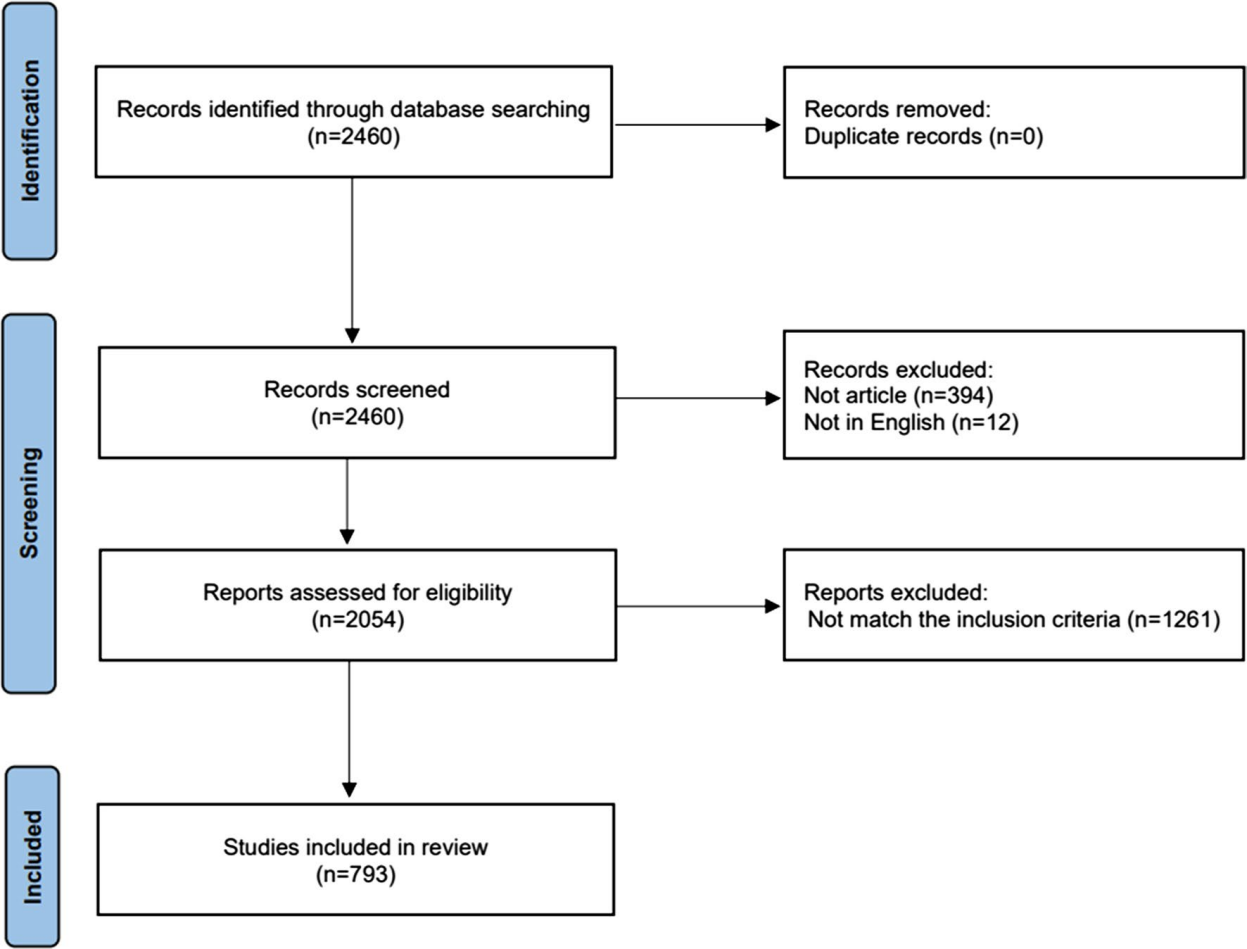
Flowchart of the literature screening process

As shown in Fig. 1, a total of 2460 publications were identified from the WoSCC database by searching for keywords related to GC and nanomaterials, and no duplicates were discovered. At the first selection stage, 12 publications were excluded due to language restriction, and 394 publications were excluded due to publication types. The titles and abstracts of the remaining 2054 publications were carefully evaluated. Finally, 793 studies meeting the inclusion criteria of this study were included.

**Fig. 2:**
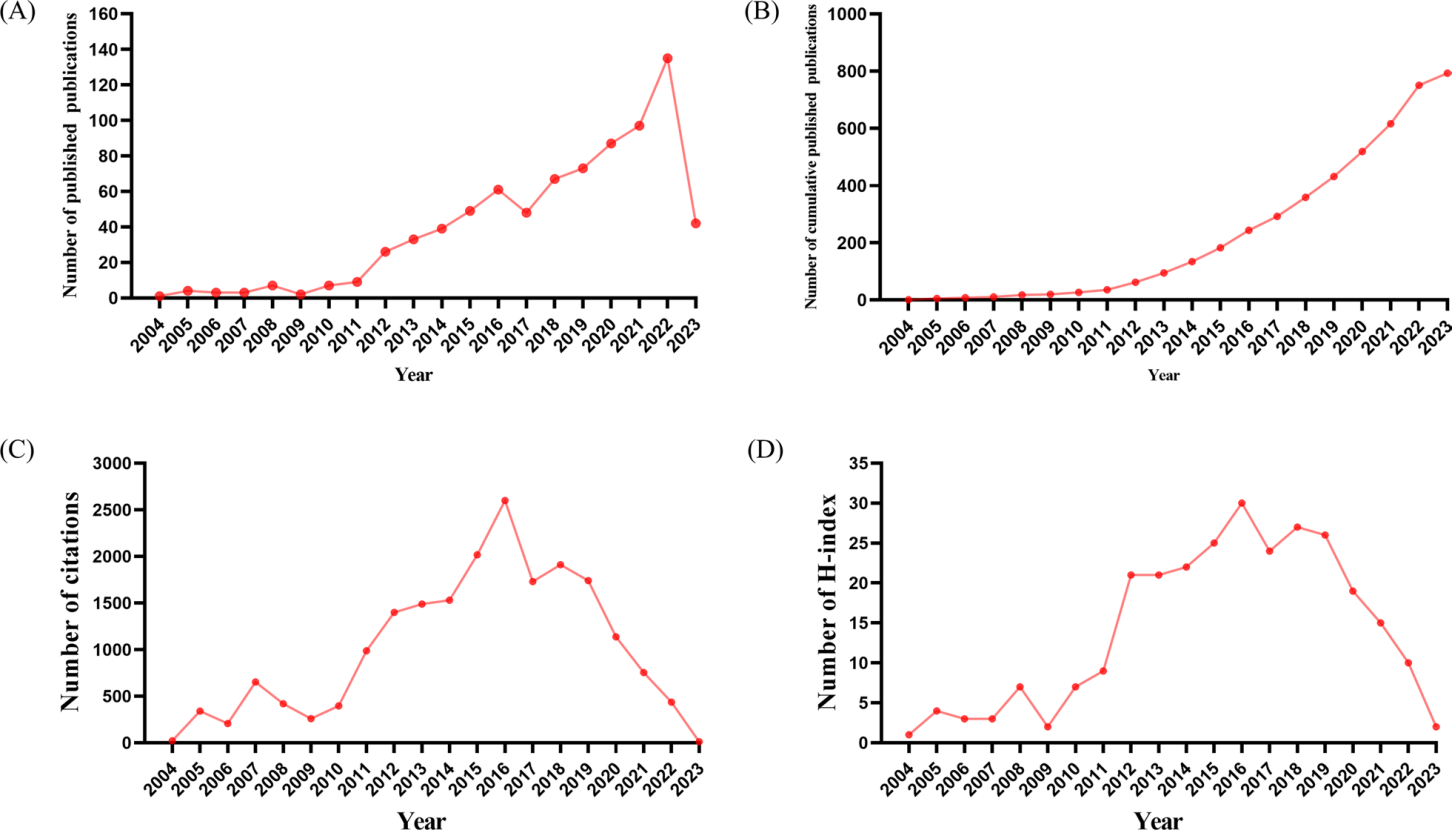
**A** The global annual number of publications; **B** the global annual number of cumulative publications; **C** the global annual number of citations of the publications; **D** the global annual H-index values of the publications

Next, we summarized the characteristics of the included studies. The distribution of annual publication numbers from 2004 to 2023 is shown in Fig. 2A. The number of annual publications showed an overall increasing trend, indicating that attention to the field of GC and nanomaterials increased. The publication number reached its peak in 2022 with 135 publications, accounting for 17.02% of the total publications. The cumulative number of publications has grown steadily from 2004 to 2023 (Fig. 2B). The number of citations was relatively high from 2014 to 2019, with over 1500 citations per year (Fig. 2C). The annual H-index increased from 1 in 2004 to 30 in 2016 (Fig. 2D).

### Analysis of country/region and institution attributes of the publications

**Fig. 3:**
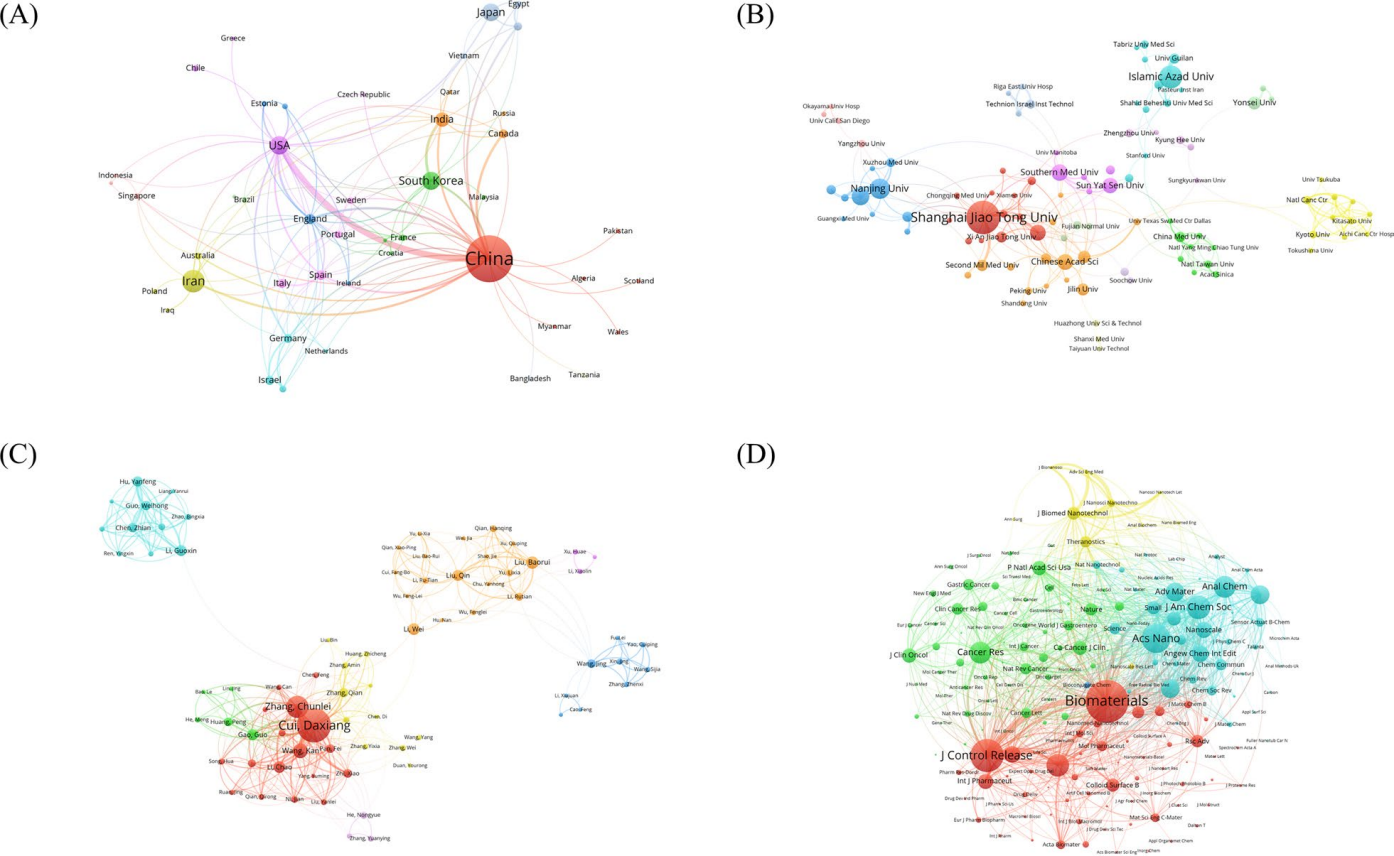
The coauthorship network map of countries **A**, institutions **B**, and authors **C**. **D** The cocitation network map of journals

The coauthorship network visualization map of countries is shown in Fig. 3A. In total, 46 countries/regions and 130 cooperation instances were presented. China had the strongest international cooperation network (TLS = 101) and cooperated most closely with the USA (LS = 29) **(**Fig. 3A**)**. Next, we analyzed the number of publications, total citations and H-index of the 10 most productive countries/regions. As shown in Table 1, China had the most publications (524, 66.08%), followed by Iran (91, 11.48%) and the USA (57, 7.19%). In addition, China had the highest number of citations (13,251) and the highest H-index (73).

**Table 1:** The top 10 most productive countries regarding nanomaterial and GC research from 2004 to 2023

Rank	Country	Counts	Percentage	Total citations	H-Index
1	China	524	66.08	13,251	73
2	Iran	91	11.48	1339	22
3	USA	57	7.19	2286	26
4	South Korea	54	6.81	1129	20
5	Japan	51	6.43	1976	22
6	India	32	4.04	472	12
7	England	14	1.77	602	8
8	Spain	13	1.64	832	10
9	Israel	11	1.39	800	7
10	Italy	11	1.39	418	6

The coauthorship network map of institutions is shown in Fig. 3B, which includes 99 institutions and 244 cooperation instances. Shanghai Jiao Tong University had the largest cooperative network (TLS = 45). The top 12 most productive institutions are shown in Table 2. Shanghai Jiao Tong University had the most publications (70, 8.83%), followed by Islamic Azad University (39, 4.92%), and Nanjing University (35, 4.41%). Shanghai Jiao Tong University also had the highest number of total citations (3337) and the highest H-index (34).

**Table 2:** The top 12 most productive institutions regarding nanomaterial and GC research from 2004 to 2023

Rank	Institution	Country	Counts	Percentage	Total citations	H-Index
1	Shanghai Jiao Tong University	China	70	8.83	3337	34
2	Islamic Azad University	Iran	39	4.92	427	10
3	Nanjing University	China	35	4.41	1275	19
4	Chinese Academy of Sciences	China	33	4.16	1025	18
5	Nanjing Medical University	China	30	3.78	818	18
6	Southern Medical University China	China	23	2.90	497	11
7	Fudan University	China	22	2.77	558	11
8	Sun Yat Sen University	China	21	2.65	461	13
9	Naval Medical University	China	16	2.02	543	11
10	University of California System	USA	16	2.02	982	11
11	Yonsei University	Korea	16	2.02	393	9
12	Wuhan University	China	16	2.02	646	9

### Analysis of authors of publications

In total, 4555 authors contributed to all the publications analyzed. The top 10 authors with the most publications are shown in Table 3. Cui DX had the highest number of publications in this field (48), followed by Zhang CL (27) and Wang K (20), all from Shanghai Jiao Tong University (China). In addition, Cui DX was the author with the highest number of total citations and the highest H-index. The author cooperation network map is shown in Fig. 3C. Cui DX had the highest number of collaborative relationships with other authors (TLS = 201).

**Table 3:** The top 10 most productive authors regarding nanomaterial and GC research from 2004 to 2023

Rank	Authors	Counts	Percentage	Total citations	H-Index
1	Cui DX	48	6.05	2855	30
2	Zhang CL	27	3.41	1967	21
3	Wang K	20	2.52	1108	15
4	Liu BR	18	2.27	647	13
5	Wang J	17	2.14	556	9
6	Wang Y	17	2.14	377	7
7	Li C	15	1.89	734	13
8	Li RT	14	1.77	522	11
9	Zhang Y	14	1.77	193	6
10	Li W	13	1.64	117	7

### Analysis of source journals and cocited journals

A total of 185 articles were published in the top 10 journals (Table 4), accounting for 23.33% of all publications. The International Journal of Nanomedicine, Journal of Biomedical Nanotechnology and Biomaterials were the top 3 journals for publishing research in this field. Biomaterials had the highest number of citations and the highest average number of citations per paper. The International Journal of Nanomedicine had the highest H-index (22), followed by Biomaterials (15). Moreover, the IF of a journal is an important parameter used to evaluate its value and that of its included publications [17]. ACS Nano had the highest IF (17.1), followed by Biomaterials (14.0). The journal cocitation network map is shown in Fig. 3D. The top 3 cocited journals were as follows: Biomaterials (846 citations), Journal of Controlled Release (638 citations) and ACS Nano (556 citations).

**Table 4:** The top 10 most productive journals regarding nanomaterial and GC research from 2004 to 2023

Rank	Journal title	Records	Total citations	Average citation	H-Index	IF (2022)
1	International Journal of Nanomedicine	41	1256	30.63	22	8
2	Journal of Biomedical Nanotechnology	21	472	22.48	10	2.9
3	Biomaterials	16	1578	98.63	15	14
4	Journal of Nanobiotechnology	13	390	30.00	9	10.2
5	ACS Applied Materials Interfaces	12	391	32.58	9	9.5
6	Analytical Chemistry	11	470	42.73	7	7.4
7	Nanoscale Research Letters	11	383	34.82	10	\
8	ACS Nano	10	867	86.70	10	17.1
9	Artificial Cells Nanomedicine and Biotechnology	10	282	28.20	8	5.8
10	International Journal of Pharmaceutics	10	309	30.90	7	5.8

### Analysis of highly cited studies

**Fig. 4:**
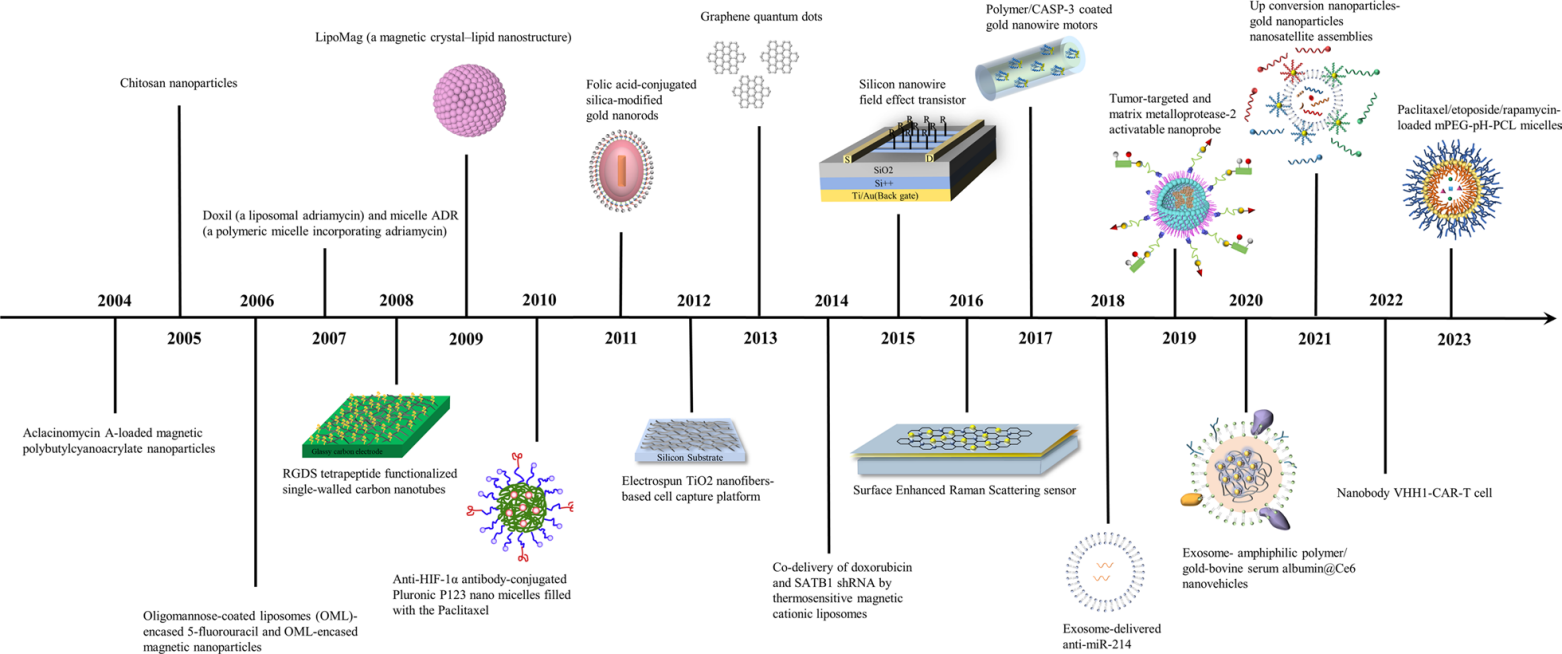
The timeline of nanomaterial and GC research

The nanomaterials analyzed in the most cited publications of each year during the study period are shown in Fig. 4 [18–37]. Gold nanomaterials were frequently included in these studies. The 10 most cited studies are shown in detail in Table 5. Three of the ten studies were from Shanghai Jiao Tong University (China), two were from National Cancer Center (Japan), and the others were from University of Tokyo (Japan), Wuhan University (China), Jikei University (Japan), Technion Israel Institution of Technology (Israel), and Anhui Medical University (China). Specifically, a study entitled “Improvement of cancer-targeting therapy, using nanocarriers for intractable solid tumors by inhibition of TGF-beta signaling” published in Proceedings of the National Academy of Sciences of the United States of America in 2007 was cited 349 times, making it the most cited publication in the field. Regarding research topics, 2 studies focused on the use of breath analysis with nanomaterial-based sensors for noninvasive diagnosis of GC, 2 focused on the use of nanomaterials for in vivo imaging and targeted photothermal therapy/photodynamic therapy, 2 focused on phase I and phase II studies of NK105, a paclitaxel-incorporating micellar NP for GC patients, 2 focused on nanocarriers delivering drugs or genes to treat cancer, 1 focused on nanomaterial-based detection of circulating tumor cells, and 1 focused on cellular internalization and cytotoxicity of graphene quantum dots [21, 23, 25–27, 29, 38–41].

**Table 5:** The top 10 most cited references regarding nanomaterial and GC research from 2004 to 2023

Rank	Title	Institution	Authors	Journal	Citations
1	Improvement of cancer-targeting therapy, using nanocarriers for intractable solid tumors by inhibition of TGF-beta signaling	University of Tokyo, Japan	Kano M, Bae Y, Iwata C, et al.	Proceedings of the National Academy of Sciences of the United States of America	349
2	Folic acid-conjugated Silica-modified gold nanorods for X-ray/CT imaging-guided dual-mode radiation and photo-thermal therapy	Shanghai Jiao Tong University, China	Huang P, Bao L, Zhang C, et al.	Biomaterials	337
3	Electrospun TiO_2_ Nanofiber-Based Cell Capture Assay for Detecting Circulating Tumor Cells from Colorectal and Gastric Cancer Patients	Wuhan University, China	Zhang N, Deng Y, Tai Q, et al.	Advanced Materials	297
4	Photosensitizer-conjugated magnetic nanoparticles for in vivo simultaneous magnetofluorescent imaging and targeting therapy	Shanghai Jiao Tong University, China	Huang P, Li Z, Lin J, et al.	Biomaterials	230
5	A phase I and pharmacokinetic study of NK105, a paclitaxel-incorporating micellar nanoparticle formulation	National Cancer Center, Japan	Hamaguchi T, Kato K, Yasui H, et al	British Journal of Cancer	220
6	A novel magnetic crystal-lipid nanostructure for magnetically guided in vivo gene delivery	Jikei University, Japan	Namiki Y, Namiki T, Yoshida H, et al.	Nature Nanotechnology	208
7	Ultrasensitive Silicon Nanowire for Real-World Gas Sensing: Noninvasive Diagnosis of Cancer from Breath Volatolome	Technion Israel Institute of Technology, Israel	Shehada N, Brönstrup G, Funka K, Christiansen S, Leja M, Haick H	Nano Letters	180
8	Phase II study of NK105, a paclitaxel-incorporating micellar nanoparticle, for previously treated advanced or recurrent gastric cancer	National Cancer Center, Japan	Kato K, Chin K, Yoshikawa T, et al.	Investigational New Drugs	169
9	A nanomaterial-based breath test for distinguishing gastric cancer from benign gastric conditions	Anhui Medical University, China	Xu Z q, Broza YY, Ionsecu R, et al.	British Journal of Cancer	167
10	Insight into the Cellular Internalization and Cytotoxicity of Graphene Quantum Dots	Shanghai Jiao Tong University,China	Wu C, Wang C, Han T, Zhou X, Guo S, Zhang J	Advanced Healthcare Materials	166

### Keyword analysis of research hotspots

**Fig. 5:**
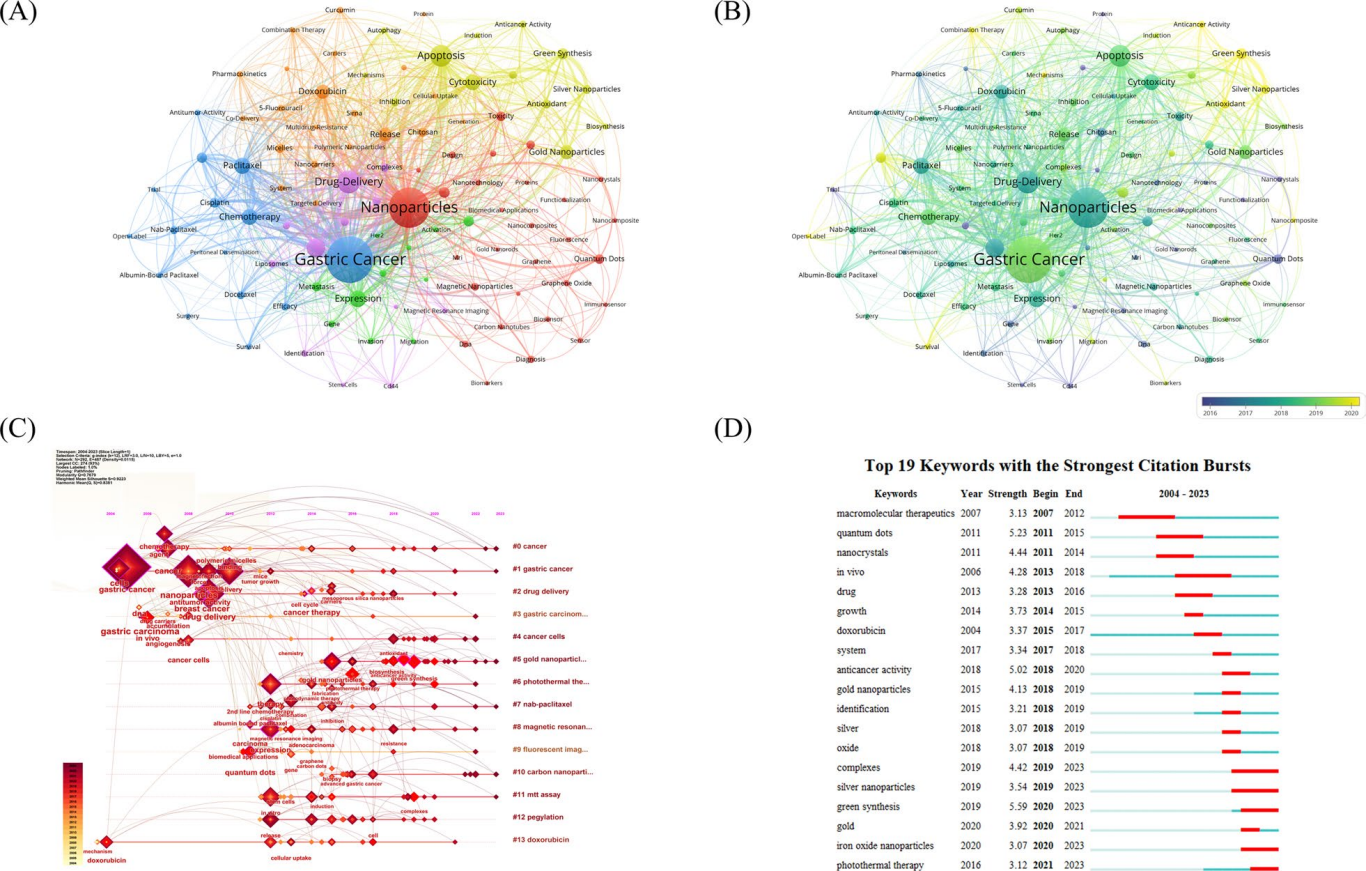
The network **A**, overlay **B**, and timeline **C** map of keyword co-occurrence. **D** The top 19 keywords with the strongest citation bursts

Keyword co-occurrence analysis is a common method used to identify popular research topics. The network and overlay visualization maps of cooccurring keywords are shown in Fig. 5A and B. The top 10 most frequently used keywords were gastric cancer, nanoparticles, drug-delivery, apoptosis, therapy, paclitaxel, chemotherapy, doxorubicin, expression and cytotoxicity. Figure 5A shows all keywords grouped into 6 clusters. The largest cluster was red and related to diagnosis and treatment of NPs, including keywords such as “nanoparticles”, “biomarkers”, “biosensor”, and “photodynamic therapy”. The second largest cluster was orange and was related to nanocarriers and loaded agents, including keywords such as “doxorubicin”, “release”, “chitosan”, and “siRNA”. The third major group was blue and was related to clinical trials of NPs, and included keywords such as “gastric cancer”, “chemotherapy”, “paclitaxel”, and “cisplatin”. The fourth group was yellow and represented the green synthesis and anticancer mechanisms of nanomaterials and included keywords such as “apoptosis”, “cytotoxicity”, “green synthesis”, and “antioxidant”. The purple cluster related to delivery and imaging, including keywords such as “drug-delivery”, “gene delivery”, “identification”, and “magnetic resonance imaging”. The green cluster was associated with tumor progression, and included keywords such as “expression”, “metastasis”, “growth”, and “activation”. As shown in Fig. 5B, terms marked in purple indicate that their average year of publication was 2016 or earlier, while those marked in bright yellow appeared after 2020. Keywords such as “quantum dots”, “nanocrystals”, “gold nanorods” and “magnetic resonance imaging” were the main topics during the early stage. The keywords “green synthesis”, “silver nanoparticles”, “antioxidant” and “anticancer activity” appeared relatively late in the study period.

In addition, we presented a visualization of the keyword evolution over time using CiteSpace (Fig. 5C). The Q-value was 0.7679, and the S-value was 0.9223 in this graph. Gastric cancer and drug delivery were the main hot research keywords before 2012. Gastric cancer, gold nanoparticle, nab-paclitaxel, carbon nanoparticle and MTT assay continue to be hot topics in 2023. Another crucial sign of the study frontiers and hotspots throughout time was the strength of the keyword bursts (Fig. 5D). Among the top 19 keywords with the strongest citation bursts, green synthesis had the highest burst strength (5.59), followed by quantum dots (5.23) and anticancer activity (5.02). Notably, the complexes, silver nanoparticles, green synthesis, iron oxide nanoparticles and photothermal therapy bursts continued in 2023, indicating that these are still hot topics.

## Discussion

To our knowledge, this was the first study to conduct a comprehensive bibliometric analysis of publications related to the application of nanomaterials in GC from 2004 to 2023. Our results showed an increasing trend in the number of annual publications in this field. 2016 was a significant year in this field because the highest number of citations occurred in this year and the highest H-index was observed. The decrease in the number of citations and H-index in the last four years might be attributed to the proximity to the time of data collection.

We analyzed the most influential countries, institutions, authors and journals in the field. China was the dominant country in terms of contributing to this field of research, as demonstrated by China having the highest number of international collaboration relationships, publications, and citations and the highest H-index. Seventy-five percent of the top 12 most productive institutions were from China. Shanghai Jiao Tong University and Cui DX were the most influential institutions and authors in the field, respectively. The International Journal of Nanomedicine, Biomaterials and ACS Nano, were considered the most influential journals in this field. The International Journal of Nanomedicine had the highest number of publications and the highest H-index; Biomaterials had the highest number of citations and the highest average citations per paper while ACS Nano had the highest journal IF.

We also analyzed the top 10 most cited publications. Highly cited studies are generally considered the most important and influential studies in the field. The most highly cited publication studied the improvement of targeted therapy for intractable solid tumors using nanocarriers. Kano et al. [21] found that low-dose small-molecule transforming growth factor β type I receptor inhibitors combined with long-circulating nanocarriers enhanced the permeability and retention effects in intractable GC, exhibited effective tumor growth inhibition, and may reduce side effects. 2 studies researched nanomaterial-based sensors for non-invasive diagnosis of GC by detecting exhaled breath. Xu et al. [38] used a nanomaterial-based sensor to analyze alveolar exhaled breath samples from 130 patients with gastric complaints and showed good discrimination between GC vs. benign gastric conditions, early stage GC (I and II) vs. late stage GC (III and IV), and ulcers vs. less severe benign conditions. Nisreen et al. [29] reported a molecularly modified silicon nanowire field effect transistor that selectively detects volatile organic compounds associated with GC in exhaled breath and validated the ability to discriminate between GC and non-GC patients. Additionally, 2 studies focused on the use of nanomaterials for simultaneous in vivo imaging and targeted photothermal therapy/photodynamic therapy. Huang et al. [25, 39] successfully designed and developed folic acid-conjugated silica-modified gold nanorods and photosensitizer chlorine e6-conjugated magnetic NPs and demonstrated their excellent imaging and targeting capabilities. 2 studies performed clinical trials with paclitaxel-incorporating micellar NP. Hamaguchi et al. [40] conducted a phase I clinical trial of NK105 to determine its maximum tolerated dose, recommended phase II dose, and pharmacokinetics, and observed an approximately 40% reduction in a GC patient with peritoneal metastases. Ken et al. [41] conducted a phase II study of NK105 for previously treated advanced or recurrent GC patients. The results showed a median progression-free survival time of 3.0 months, a median time to treatment failure of 2.8 months, a median overall survival time of 14.4 months, and no treatment-related deaths. Zhang et al. [26] demonstrated a new circulating tumor cell capture platform that reliably captures cancer cells from artificial blood samples, colorectal cancer patients, and GC patients. Namiki et al. [23] reported that a novel magnetic crystal-lipid nanostructure delivered and silenced genes in cells and tumors in mice and showed significant anti-tumor effects when administered systemically to mice with GC. Wu et al. [27] found that the toxicity of graphene quantum dots to GC cells was lower than that of micrometer-sized graphene oxide, and the low cytotoxicity and size consistency of graphene quantum dots could be employed as carriers for targeted drug delivery.

The keyword analysis showed that the most frequently occurring keywords were related to drug delivery and anticancer mechanisms, indicating that they were the most widely researched subfields. In the network visualization diagram, all keywords were divided into the following six clusters: diagnosis and treatment, nanocarriers and load agents, clinical trials, green synthesis and anti-cancer mechanisms, delivery and imaging, and tumor progression. These six clusters demonstrated the main topics explored in the research area. From the overlay map, we found that keywords related to diagnosis and treatment emerged early, while green synthesis and anticancer mechanisms were recent research hotspots.

Nanomaterials have multiple advantages and face many challenges when used in GC applications. The nanomaterial applications overcome some of the shortcomings of traditional diagnostic methods, such as endoscopy, tumor marker detection, computed tomography and magnetic resonance imaging. For example, in endoscopy, surface-enhanced Raman scattering NPs can increase the Raman signal intensity while combining with corresponding target ligands to diagnose early-stage tumors and differentiated lesion tissues; in tumor marker detection, gold NPs, silicon nanowires, and quantum dots can improve the sensitivity and accuracy of tumor marker detection in serum or tissue samples; and in tumor imaging, superparamagnetic iron oxide nanoparticles (SPION), gold NPs and nanoprobes play important roles in improving targeting, biocompatibility and bioavailability. [2, 42] Gold NPs, magnetic NPs, quantum dots and TiO_2_ NPs are expected to enhance the detection of circulating tumor cells in the blood and play a role in the diagnosis and prognosis of metastatic GC. [42]

Nanomaterials have many advantages in phototherapy, chemotherapy, targeted therapy and combination therapy for GC. The addition of nanomaterials improves the responsive release, tissue penetration depth and precise targeting of phototherapy, providing precise treatment for specific cancer tissues and cells through photodynamic therapy, photothermal therapy and combination therapy; multifunctional modified nanotechnology reduces the problems of poor solubility and bioavailability, systemic side effects and chemotherapeutic drug resistance development; the advantages of efficient loading and responsive release based on nanocarriers compensate for the insufficiently controlled release and drug resistance generation of targeted therapy; nanomaterials can be used in surgery navigation to clearly identify the location and the edge of the tumor, as well as metastatic lymph nodes for accurate resection [2, 43]. The effectiveness of immune checkpoint blockade therapy cannot be significantly improved with the use of NPs as drug delivery carriers, but in combination with chemotherapy and other modalities, NPs can not only improve the efficiency of drug delivery and utilization but also enhance the anticancer immune response [44].

In addition, the use of a variety of nanomaterials for the simultaneous diagnosis and treatment of GC has been reported. Some NP frameworks with inherent imaging capabilities, such as gold NPs for computed tomography and SPIONs for magnetic resonance imaging, are excellent candidates for use in theranostic system construction; most photosensitizers, such as IR780 and chlorine e6, have imaging capabilities and tumor toxicity, and it is also possible to efficiently load both diagnostic and therapeutic drugs into the same NPs [5].

However, most of the nanomaterial products in nanomedicine are still in the stage of in vitro cell culture or in vivo animal experiments, but the realization of clinical application still remains many challenges. The main reasons include the following: the low transfer efficiency of NPs to tumors and the lack of understanding of the molecular mechanisms of ions acting on living cells; technical challenges, such as the synthesis of NPs, and the excellent properties of NPs; and the great heterogeneity between human diseases and basic experimental models, resulting in a low probability of translation into human clinical research [2]. In vivo biological system efficacy and safety evaluation system has not yet been established. For example, the ultra-small size is conducive to better clearance by the kidneys, but the optimal concentration of NPs in plasma, renal clearance and residual accumulation in the body need to be quantified by safety standards. Moreover, whether the properties of nanomaterial products are likely to cause acute or chronic adverse effects in humans and whether they are caused by the drug itself or by the NPs used as drug carriers should be determined; the distribution and metabolism of nanomaterials in the human body should be understood to overcome issues caused by accidental overdosage, misuse, or accumulation of nanomaterials; and a multidisciplinary team of researchers in chemistry, materials, toxicology, biology, zoology, basic and clinical medicine should collaborate to build a bridge between the laboratory and the clinic [45].

Our study also had some limitations. First, the publications were only derived from the WoSCC database, which might have led to an incomplete literature search. However, the WoSCC database is one of the most extensive and comprehensive global databases and the most commonly used source of publications for bibliometric analysis. The data from WoSCC are large enough to reflect the current state of research in the field. Second, we only selected studies published in English.

In conclusion, we used various statistical software programs for bibliometric analysis to obtain an overview of the application of nanomaterials in GC diagnosis and treatment. We demonstrated the characteristics of publications, identified the most influential countries, institutions, authors and journals, and indicated research hotspots and trends in the field of nanomaterial use for GC diagnosis and treatment. Furthermore, we discussed the advantages and challenges faced by nanomaterials in the diagnosis and treatment of GC. Nanomaterials could be a powerful tool for the diagnosis and treatment of GC.

## Data Availability

Data sharing is not applicable to this article, as no datasets were generated or analyzed in the current study.
